# Large-Scale Metagenome Assembly Reveals Novel Animal-Associated Microbial Genomes, Biosynthetic Gene Clusters, and Other Genetic Diversity

**DOI:** 10.1128/mSystems.01045-20

**Published:** 2020-11-03

**Authors:** Nicholas D. Youngblut, Jacobo de la Cuesta-Zuluaga, Georg H. Reischer, Silke Dauser, Nathalie Schuster, Chris Walzer, Gabrielle Stalder, Andreas H. Farnleitner, Ruth E. Ley

**Affiliations:** a Department of Microbiome Science, Max Planck Institute for Developmental Biology, Tübingen, Germany; b TU Wien, Institute of Chemical, Environmental, and Bioscience Engineering, Research Group for Environmental Microbiology and Molecular Diagnostics, Vienna, Austria; c ICC Interuniversity Cooperation Centre Water and Health, Vienna, Austria; d Research Institute of Wildlife Ecology, University of Veterinary Medicine, Vienna, Austria; e Wildlife Conservation Society, Bronx, New York, USA; f Research Division Water Quality and Health, Karl Landsteiner University for Health Sciences, Krems an der Donau, Austria; University of California San Diego

**Keywords:** animal microbiome, gut, metagenome assembly, novel diversity, antimicrobial resistance, biosynthetic gene cluster, vertebrate-microbe

## Abstract

Large-scale metagenome assemblies of human microbiomes have produced a vast catalogue of previously unseen microbial genomes; however, comparatively few microbial genomes derive from other vertebrates. Here, we generated 5,596 metagenome-assembled genomes (MAGs) from the gut metagenomes of 180 predominantly wild animal species representing 5 classes, in addition to 14 existing animal gut metagenome data sets. The MAGs comprised 1,522 species-level genome bins (SGBs), most of which were novel at the species, genus, or family level, and the majority were enriched in host versus environment metagenomes. Many traits distinguished SGBs enriched in host or environmental biomes, including the number of antimicrobial resistance genes. We identified 1,986 diverse biosynthetic gene clusters; only 23 clustered with any MIBiG database references. Gene-based assembly revealed tremendous gene diversity, much of it host or environment specific. Our MAG and gene data sets greatly expand the microbial genome repertoire and provide a broad view of microbial adaptations to the vertebrate gut.

**IMPORTANCE** Microbiome studies on a select few mammalian species (e.g., humans, mice, and cattle) have revealed a great deal of novel genomic diversity in the gut microbiome. However, little is known of the microbial diversity in the gut of other vertebrates. We studied the gut microbiomes of a large set of mostly wild animal species consisting of mammals, birds, reptiles, amphibians, and fish. Unfortunately, we found that existing reference databases commonly used for metagenomic analyses failed to capture the microbiome diversity among vertebrates. To increase database representation, we applied advanced metagenome assembly methods to our animal gut data and to many public gut metagenome data sets that had not been used to obtain microbial genomes. Our resulting genome and gene cluster collections comprised a great deal of novel taxonomic and genomic diversity, which we extensively characterized. Our findings substantially expand what is known of microbial genomic diversity in the vertebrate gut.

## Abstract (precis)

Microbiome studies on a select few mammalian species (e.g., humans, mice, and cattle) have revealed a great deal of novel genomic diversity in the gut microbiome. However, little is known of the microbial diversity in the gut of other vertebrates. We studied the gut microbiomes of a large set of mostly wild animal species consisting of mammals, birds, reptiles, amphibians, and fish. Unfortunately, we found that existing reference databases commonly used for metagenomic analyses failed to capture the microbiome diversity among vertebrates. To increase database representation, we applied advanced metagenome assembly methods to our animal gut data and to many public gut metagenome data sets that had not been used to obtain microbial genomes. Our resulting genome and gene cluster collections comprised a great deal of novel taxonomic and genomic diversity, which we extensively characterized. Our findings substantially expand what is known of microbial genomic diversity in the vertebrate gut.

## INTRODUCTION

The vertebrate gut microbiome comprises a vast amount of genetic diversity, yet even for the most well-studied species, such as humans, the number of microbial species lacking a reference genome was recently estimated to be 40 to 50% (1). Uncovering this “microbial dark matter” is essential for understanding the roles of individual microbes, their intra- and interspecies diversity within and across host populations, and how each microbe interacts with each other and the host to mediate host physiology in a myriad number of ways (2). On a more applied level, characterizing novel gut microbial diversity aids in the bioprospecting of novel bioactive natural products, catalytic and carbohydrate-binding enzymes, and probiotics, etc., along with aiding in the discovery and tracking of novel pathogens and antimicrobial resistance (AMR) (3).

Recent advances in culturomic approaches have generated thousands of novel microbial genomes (4–6), but the throughput is currently far outpaced by metagenome assembly approaches (7). However, such large-scale metagenome assembly-based approaches have not been as extensively applied to most nonhuman vertebrates. The small amount of metagenome reads classified in some recent studies of the rhinoceros, chicken, cod, and cow gut/rumen microbiomes suggests that databases lack much of the genomic diversity in less studied vertebrates (8–11). Indeed, the limited number of studies incorporating metagenome assembly hint at the extensive amounts of as-yet-novel microbial diversity across the >66,000 vertebrate species on our planet.

Here, we developed an extensive metagenome assembly pipeline and applied it to a multispecies data set of microbiome diversity across vertebrate species comprising 5 classes, Mammalia, Aves, Reptilia, Amphibia, and Actinopterygii, with >80% of samples obtained from wild individuals (12), combined with data from 14 published animal gut metagenomes. Moreover, we also applied a recently developed gene-based metagenome assembly pipeline to the entire data set in order to obtain gene-level diversity for rarer taxa that would otherwise be missed by genome-based assembly (13, 14). Our assembly approaches yielded a great deal of novel genetic diversity, which we found to be largely enriched in animals versus the environment and, to some degree, enriched in particular animal clades.

## RESULTS

### Animal gut metagenomes from a highly diverse collection of animals.

**FIG 1:**
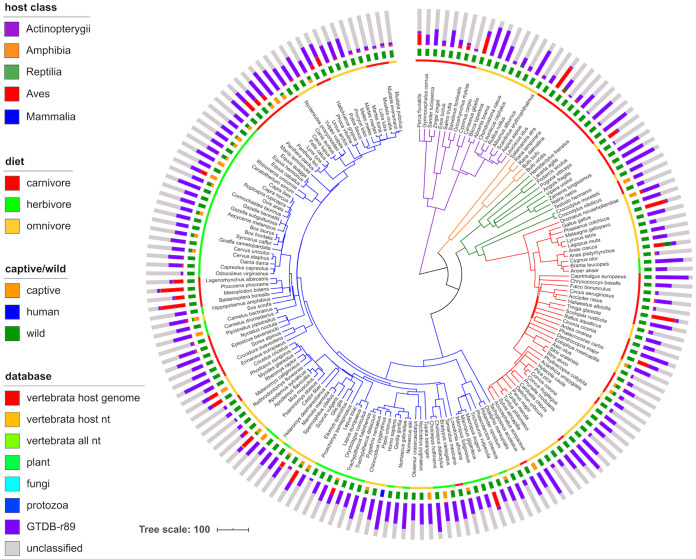
Large percentage of unmapped reads, even when using multiple comprehensive metagenome profiling databases. The dated host species phylogeny was obtained from http://timetree.org, with branches colored by host class. From inner to outer rings, the data mapped onto the tree are host diet, host captive/wild status, and mean number of metagenome reads mapped to various host-specific, nonmicrobial, and microbial databases. Note that captive/wild status sometimes differs among individuals of the same species. The databases are (i) representative of each publicly available genome from the host species (“vertebrata host genome”), (ii) all entries in the NCBI nucleotide (nt) database with taxonomy identifications matching host species (“vertebrata host nt”), (iii) the same as the previous category but with all Vertebrata sequences included, (iv) the Kraken2 “plant” database, (v) the Kraken2 “fungi” database, (vi) the Kraken2 “protozoa” database, and (vii) a custom bacterial and archaeal database created from the Genome Taxonomy Database, release 89 (“GTDB-r89”). Reads were mapped iteratively to each database in the order shown in the key (top to bottom), with only unmapped reads included in the next iteration. “Unclassified” reads did not map to any database, which were used along with reads mapping to GTDB-r89 for downstream analyses (“microbial + unclassified”).

We generated animal gut metagenomes from a breadth of vertebrate diversity spanning five classes: Mammalia, Aves, Reptilia, Amphibia, and Actinopterygii (the “multispecies” data set) (Fig. 1). In total, 289 samples passed our read quality control (QC), with 3.4e6 ± 5e6 (standard deviation [SD]) paired-end reads per sample, resulting in a mean estimated coverage ± SD of 0.54 ± 0.14 (see Fig. S1 in reference 15). One hundred eighty animal species were represented, with up to 6 individuals per species (mean of 1.6). Most individuals were wild (81%).

Our read quality control pipeline included stringent filtering of host reads; some samples contained large amounts of reads mapping to vertebrate genomes (up to 74%; 6% ± 17% SD) (Fig. 1). Gut content samples contained a significantly large amount of host reads (13.5% ± 21.6% SD) versus feces metagenomes (4.7% ± 12.7% SD; Wilcox *P* value of <1.8e−7) (see Table S1A in the supplemental material). We mapped all remaining reads to a custom comprehensive Kraken2 database built from the Genome Taxonomy Database, release 89 (GTDB-r89). Still, many samples had a low percentage of mapped reads (43% ± 22% SD) (Fig. 1), with 29% of the samples having <20% mapped reads.

10.1128/mSystems.01045-20.1 TABLE S1 (A) Metadata for all samples in the multispecies metagenome data set. (B) Summary of NCBI BioProjects used for the “multistudy” metagenome assemblies. “Number of samples used” indicates the number of metagenome samples used for metagenome assemblies. Data sets with >100 samples were randomly subsampled to 100. Metagenome assemblies were performed on a per-sample basis, with contig binning (generation of MAGs) performed on a per-study basis. Samples under BioProject accession numbers PRJNA316560 and PRJNA316570 were combined due to low numbers of samples and overlap in the animal hosts, methods, and study authors. There is no publication associated with BioProject accession number PRJEB23642. (C) All samples obtained from the MGnify database in order to create the “host-environment” metagenome data set. (D) Genome assembly accession numbers for all host species genomes used to filter out reads mapping to those genomes (the “vertebrata host genome” Kraken2 database shown in Fig. 1). (E) Software parameters used. Download Table S1, XLSX file, 0.05 MB. Copyright © 2020 Youngblut et al. 2020 Youngblut et al. This content is distributed under the terms of the Creative Commons Attribution 4.0 International license.

### Discovery of novel diversity by large-scale metagenome assembly.

Our comprehensive metagenome assembly pipeline generated 4,374 nonredundant metagenome-assembled genomes (MAGs). After quality control and dereplication (see Materials and Methods), 296 MAGs remained, with mean percent completeness and contamination ± SD of 84% ± 14% and 1.5% ± 1.2%, respectively (see Fig. S2 and supplemental results in reference 15).

We expanded our MAG data set by applying our assembly pipeline to 14 publicly available animal gut metagenome data sets in which no MAGs have been generated by *de novo* metagenome assembly (Table S1B). Our metagenome selection included 554 samples from members of the Mammalia (dogs, cats, woodrats, pigs, whales, rhinoceroses, pangolins, and nonhuman primates), Aves (geese, kakapos, and chickens), and Actinopterygii (cod). We applied our assembly pipeline to each individual data set and generated a total of 5,301 nonredundant MAGs (see Fig. S3 and supplemental results in reference 15). The substantially higher number of MAGs from these 14 data sets than in our single multispecies data set is likely due to the larger number of samples and the high sequencing depth for many of those samples (e.g., we used 2 billion paired-end reads from the dog gut microbiome data set [16]).

**FIG 2:**
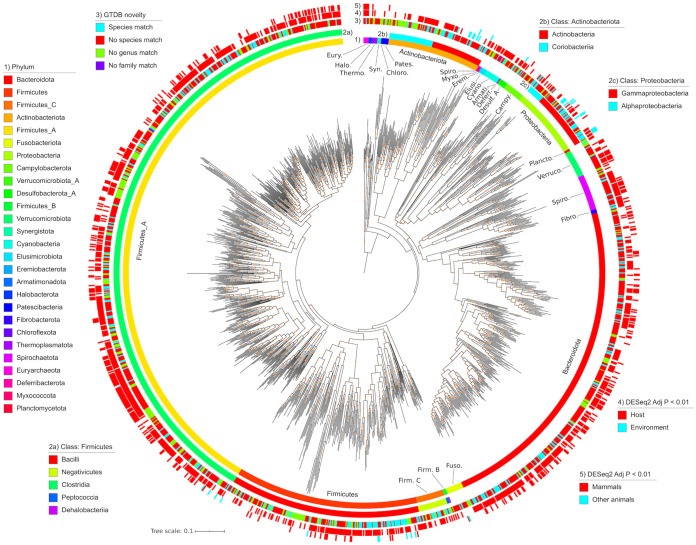
A phylogeny of all 1,522 SGBs. From innermost to outermost rings, the data mapped onto the phylogeny are GTDB phylum-level taxonomic classifications, class-level taxonomies for *Actinobacteriota*, class-level taxonomies for *Firmicutes*, class-level taxonomies for *Proteobacteria*, taxonomic novelty, significant enrichment in host gut or environmental metagenomes, and significant enrichment in mammals or other animals in our multispecies gut metagenome data set. The phylogeny was inferred from multiple conserved loci via PhyloPhlAn. Orange dots on the phylogeny denote bootstrap values in the range of 0.7 to 1. The phylogeny is rooted on the last common ancestor of *Archaea* and *Bacteria*.

We combined all MAGs and dereplicated at 99.9 and 95% average nucleotide identities (ANIs) to produce 5,596 nonredundant MAGs and 1,522 species-level genome bins (SGBs), respectively (Table S2A and B). Of the 5,596 MAGs, 2,773 (50%) had a completeness of ≥90%. Of the 1,522 SGBs, 1,184 (78%) lacked a ≥95% ANI match to GTDB-r89, 266 (17%) lacked a genus-level match, and 6 lacked a family-level match (Fig. 2) (see Fig. S4 in reference 15). Mapping taxonomic novelty onto a multilocus phylogeny of all 1,522 SGBs revealed that novel taxa were rather dispersed across the phylogeny (Fig. 2).

**FIG 3:**
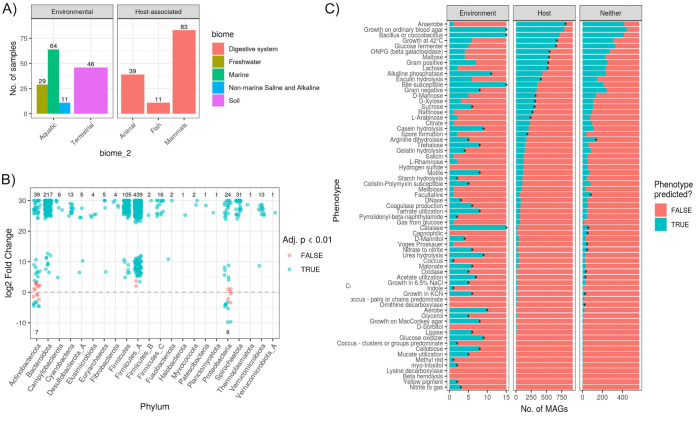
(A) Summary of the number of samples per biome for our multienvironment metagenome data set selected from the MGnify database. (B) Number of SGBs found to be significantly enriched in relative abundances in host (positive log_2_-fold change [“l2fc”]) versus environmental (negative l2fc) metagenomes. Values shown are the number of MAGs significantly enriched (blue) in either biome or not found to be significant (red). (C) Host- and environment-enriched SGBs have distinct traits. Phenotypes predicted based on MAG gene content (via Traitar [26]) are summarized for the SGBs significantly enriched in host or environmental metagenomes (DESeq2 adjusted *P* value of <0.01) or neither biome (“Neither” in the *x* axis facet). Note the difference in the *x* axis scale. Asterisks denote phenotypes significantly more prevalent in SGBs of the particular biome than in a null model of 1,000 permutations in which biome labels were shuffled among SGBs. See Table S3A in the supplemental material for all DESeq2 results. ONPG, *o*-nitrophenyl-β-d-galactopyranoside.

10.1128/mSystems.01045-20.2 TABLE S2 (A) Metadata on all nonredundant (dereplication at 99.9% ANI), quality (CheckM-estimated completeness of ≥50% and contamination of <5%) MAGs from all metagenome assemblies (those generated in this study and those reported previously). Taxonomy and ANI values were derived from GTDB-Tk. (B) Metadata on all SGBs. Genome assembly metrics refer to the SGB reference genome. (C) Traitar-derived phenotype predictions for SGBs grouped by phylum and the biome for which they were enriched (host, environment, or neither) (Fig. 3C). Download Table S2, XLSX file, 0.6 MB. Copyright © 2020 Youngblut et al. 2020 Youngblut et al. This content is distributed under the terms of the Creative Commons Attribution 4.0 International license.

We also assessed the novelty of our SGBs relative to the UHGG, a comprehensive human gut genome database, and found that only 31% of our SGBs had ≥95% ANI to any of the 4,644 UHGG representatives, and this overlap increased to only 34% at a 90% ANI cutoff.

Our SGB collection mostly consisted of MAGs assembled from a few species in the multistudy data set, suggesting that the SGBs may not be representative of taxa found in other, more distantly related vertebrates. To assess the level of representation, we determined the prevalence of all SGBs across all multispecies metagenomes (see Fig. S5 in reference 15). The host species with the highest number of observed SGBs tended to be those comprising the multistudy data set (e.g., pigs and primates); however, SGBs were frequently observed across the host phylogeny (41 ± 61 [SD] SGBs per host), indicating that the SGB collection was generally representative of the vertebrate gut microbiome.

Integrating the 1,522 SGBs into our custom GTDB Kraken2 database significantly increased the percentage of reads mapped (*P < *0.005 by a paired *t* test) (see Fig. S6 in reference 15). The percent increase varied from <1 to 62.8% (mean ± SD of 5.3% ± 6.7%) among animal species but did not appear biased to just pigs, dogs, or other vertebrate species in the multistudy data sets that we incorporated (see Fig. S7 in reference 15), which corresponds with our analysis of SGB prevalence across vertebrate hosts (see Fig. S5 in reference 15).

### Enrichment of SGBs among animal clades.

While the MAGs generated here derive from animal gut metagenomes, many of these taxa might be transient in the host and actually more prevalent in the environment. We tested this by generating a “host-environment” metagenome data set comprising 283 samples from 30 BioProjects (17 environmental and 13 host associated) (Fig. 3A). We found 932 of the 1,522 SGBs (61%) to be significantly enriched in the host metagenomes (DESeq2 adjusted *P* value of <0.01) (Fig. 3B). The host-enriched SGBs (host-SGBs) were taxonomically diverse, comprising 22 phyla. In contrast, only 15 SGBs (1%) were environment enriched (env-SGBs), all of which belonged to either the *Actinobacteriota* or *Proteobacteria* (Fig. 3B). The only SGBs that were not significantly enriched in either group belonged to the *Actinobacteriota* or *Proteobacteria*, along with two SGBs from the *Firmicutes* A phylum. Mapping these data onto the SGB phylogeny revealed phylogenetic clustering of the environment-enriched SGBs (Fig. 2).

10.1128/mSystems.01045-20.3 TABLE S3 (A) DESeq2 results for testing of host versus environment enrichment of SGB abundances (inferred via Krakenuniq). Only SGBs with a prevalence of >5% were included in the analysis (*n *= 967). Positive and negative log_2_-fold change values signify enrichment in host-associated and environment metagenomes, respectively. These data are shown in Fig. 3. (B) DESeq2 results for testing of Mammalia versus non-Mammalia enrichment of SGB abundances (inferred via Krakenuniq). Only SGBs with a prevalence of >5% were included in the analysis (*n *= 663). Positive and negative log_2_-fold change values signify enrichment in Mammalia and non-Mammalia metagenomes, respectively. These data are shown in Fig. S8 of N. D. Youngblut, J. de la Cuesta-Zuluaga, G. H. Reischer, S. Dauser, et al. (bioRxiv, 2020, https://doi.org/10.1101/2020.06.05.135962). Download Table S3, XLSX file, 0.1 MB. Copyright © 2020 Youngblut et al. 2020 Youngblut et al. This content is distributed under the terms of the Creative Commons Attribution 4.0 International license.

We investigated the traits of the host- and environment-enriched SGBs and found many predicted phenotypes to be more prevalent in one group or the other (Fig. 3C; Table S2C). A total of 67 traits were predicted based on the genomic content of certain Pfam domains (17). Almost all env-SGBs were aerobes (93%), which may aid in transmission between the environment and host biomes. In contrast, 87% of host-SGBs were anaerobes. Furthermore, all env-SGBs could generate catalase and were bile susceptible, while both phenotypes were sparse in host-SGBs (Fig. 3C). Carbohydrate metabolism also differed, with most host-SGBs predicted to consume various tri-, di-, and monosaccharides. In contrast, env-SGBs were enriched in phenotypes associated with motility, nitrogen metabolism, and the breakdown of heterogeneous substrates (e.g., cellobiose metabolism).

We also compared SGB enrichment in mammals versus nonmammals in our “multispecies” metagenome data set and found 361 SGBs (24%) to be significantly enriched in mammals, while 22 (1%) were enriched in nonmammals (DESeq2 adjusted *P* value of <0.01) (see Fig. S2C in reference 15). Interestingly, 100% of SGBs in the two archaeal phyla (*Halobacteria* and *Euryarchaeota*) were enriched in mammals. Also of note, most of the *Verrucomicrobiota* SGBs (87%) were enriched in mammals. The only 2 phyla with >10% of SGBs enriched in nonmammals were *Proteobacteria* (29%) and *Campylobacteria* (25%).

In contrast to our assessment of phenotypes distinct to host- or env-SGBs, we did not observe such a distinction of phenotypes among SGBs enriched in Mammalia or nonmammal gut metagenomes (see Fig. S8 in reference 15). Certain phenotypes such as anaerobic growth and lactose consumption were more prevalent among mammal species, but they were not found to be significantly enriched relative to the null model.

Little is known about the distribution of antimicrobial resistance genes in the gut microbiomes of most vertebrate species (18); therefore, we investigated the distribution of AMR genes among MAGs enriched in the environment versus host biomes. We found a mean ± SD of 35 ± 26 AMR markers per genome (see Fig. S9A in reference 15). The high average was largely driven by *Proteobacteria* and *Campylobacter* genomes, which had means of 387 and 161 AMR markers per genome, respectively. The 5 most abundant markers were *ruvB*, *galE*, *tupC*, *fabL* (*ygaA*), and *arsT* (see Fig. S9A in reference 15). The more abundant markers predominantly belonged to *Firmicutes* A, while *Proteobacteria* comprised larger fractions of the less abundant markers. Environment-enriched taxa contained substantially more AMR genes than host-enriched taxa, and the same was true for non-Mammalia versus Mammalia-enriched taxa (see Fig. S9B and C in reference 15).

### MAGs reveal novel secondary metabolite diversity.

We identified 1,986 biosynthetic gene clusters (BGCs) among all 1,522 SGBs. A total of 28 different products were predicted, with the most abundant being nonribosomal peptide synthetases (NRPSs) (*n *= 473), sactipeptides (*n *= 307), and arylpolyenes (*n *= 291) (see Fig. S10 in reference 15). BGCs were identified in 2 archaeal and 18 bacterial phyla. MAGs in the *Firmicutes* A phylum contained the most BGCs (*n *= 764; 38%), while the *Bacteroidota* and *Actinobacteriota* phyla possessed 381 (19%) and 272 (14%) BGCs, respectively (see Fig. S10 in reference 15). Still, *Actinobacteriota* SGBs possessed the highest average number of BGCs per genome (16.3 BGCs), followed by *Eremiobacterota* (9), *Proteobacteria* (7.7), and *Halobacterota* (5.1).

**FIG 4:**
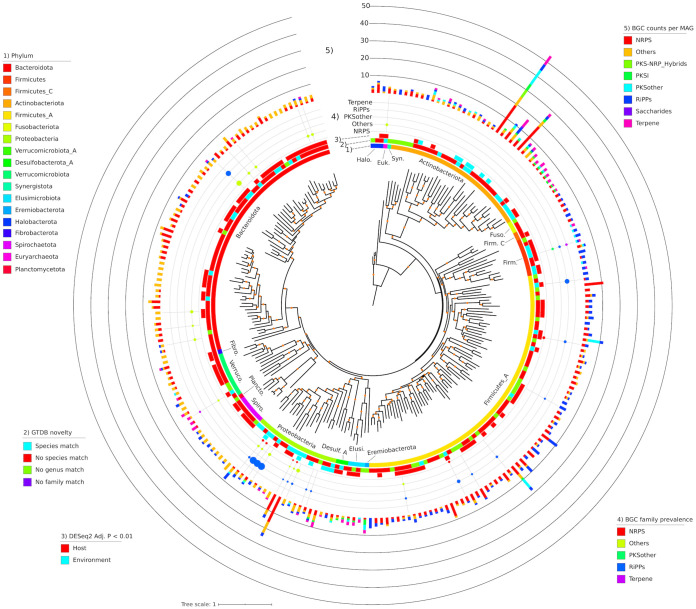
Phylogeny of all SGBs (*n *= 233) with ≥3 BGCs identified by AntiSMASH. From innermost to outermost rings, the data mapped onto the phylogeny are (i) GTDB phylum-level taxonomic classifications, (ii) taxonomic novelty, (iii) significant enrichment in host or environmental metagenomes, (iv) the prevalence of BGC families across the multispecies metagenome data set, and (v) the number of BGCs identified in the MAG. Prevalence is the maximum of any BGC family for that BGC type, and only BGC families with a prevalence of ≥25% are shown. The phylogeny is a pruned version of that shown in Fig. 2. Orange dots on the phylogeny denote bootstrap values in the range of 0.7 to 1. “NPRS”, “PKS,” and “RiPPs” stand for nonribosomal peptide synthetase, polyketide synthase, and ribosomally synthesized and posttranslationally modified peptides, respectively.

Clustering all 1,986 BGCs by BiGSCAPE generated 1,764 families and 1,305 clans, with clans being a second, coarser level of clustering (19). Only 8 clans (comprising 23 BGCs) included any MIBiG database reference, suggesting a high degree of novelty (see Fig. S11 in reference 15). Mapping the BGCs on a genome phylogeny of all species containing ≥3 BGCs (233 SGBs) revealed that the number of BGCs per genome was somewhat phylogenetically clustered: the five genomes with the most BGCs belonged to either the *Actinobacteria* or *Gammaproteobacteria* (Fig. 4). Notably, these clades contained a high number of host-SGBs. Of these 233 SGBs, the majority were taxonomically novel, with 62% lacking a species-level match to GTDB-r89 and 18% lacking a genus-level match (Fig. 4). To determine which of the BGCs are most prevalent across animal hosts, we quantified the prevalence of each BGC family across our multispecies metagenome data set and mapped it to the genome phylogeny (Fig. 4) (see Fig. S12 in reference 15). Of the 1,543 BGC families found in the 233 SGBs, 83 were present in ≥25% of the animal metagenomes, with ribosomally synthesized and posttranslationally modified peptides (RiPPs) being by far the most prevalent (up to a 98% prevalence of individual BGC families) and also found in species from a number of phyla.

### Large-scale gene-based metagenome assembly reveals novel diversity.

**FIG 5:**
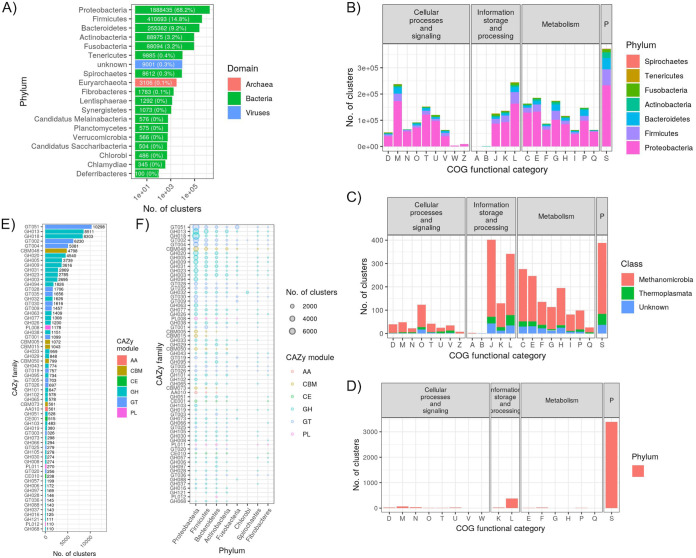
Summary of the 50% sequence identity clusters generated from the gene-based metagenome assembly of the combined data set. (A) Total number of gene clusters per phylum. For clarity, only phyla with ≥100 clusters are shown. Labels on each bar list the number of clusters (and percentage of the total). (B) Number of bacterial gene clusters per phylum and COG category. The “P” facet label refers to “poorly characterized.” (C) Number of archaeal gene clusters per class (all belonging to the *Euryarchaeota*) and COG category. (D) Number of viral gene clusters per COG category. (E) Number of clusters annotated as each CAZy family. For clarity, only phyla with ≥100 clusters are shown. Labels next to each bar denote the number of clusters. (F) Number of clusters per CAZy family, broken down by phylum. CAZy families and phyla are ordered by most to least clusters. For clarity, only CAZy families and phyla with ≥100 total clusters are shown.

We applied gene-based assembly methods to our combined metagenome data set (14), which generated a total of 150,718,125 nonredundant coding sequences (average length of 179 amino acids). Clustering at 90 and 50% sequence identities resulted in 140,225,322 and 6,391,861 clusters, respectively. Only 16.9 and 11.3% of each respective cluster set mapped to the UniRef50 database, indicating that most coding sequences were novel. The clusters comprised 88 bacterial and 11 archaeal phyla, 80 of which were represented by <100 clusters, with 60 lacking a cultured representative. *Proteobacteria* (mostly *Gammaproteobacteria*), *Firmicutes*, and *Bacteroidetes* made up 92.2% of all clusters (Fig. 5A). The proportion of clusters belonging to each Clusters of Orthologous Groups (COG) functional category was largely the same for the more abundant bacterial phyla (Fig. 5B), while more variation was seen among *Euryarchaeota* (Fig. 5C). The dominant 7 phyla showed substantial variation in the number of clusters associated with various KEGG pathway categories (see Fig. S14 in reference 15). For instance, high proportions of *Fusobacteria* and *Tenericutes* clusters were associated with the “nucleotide metabolism,” “replication and repair,” and “translation” categories. A total of 87,573 clusters were annotated as CAZy families, with GT51, GH13, GH18, GT02, and GT04 representing 48% of all CAZy-annotated clusters (Fig. 5E). Of the 12 phyla with the most CAZy family clusters, there were substantial differences in the proportions of clusters falling into each family (Fig. 5F).

### Biome enrichment of gene clusters from specific phyla.

**FIG 6:**
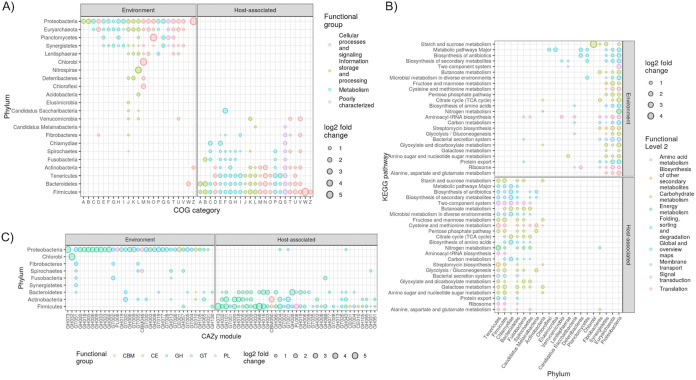
Enrichment of gene clusters grouped by phylum and COG category (A), KEGG pathway (B), or CAZy family (C). Only groupings significantly enriched in abundance (DESeq2 adjusted *P* value of <1e−5) in either biome are shown. Only gene clusters observed in at least 25% of the metagenomes were included. For clarity, only KEGG pathways enriched in >7 phyla are shown, and only CAZy families enriched in >1 phylum are shown. Note that the axes are flipped in panel B relative to panels A and C. See Tables S5A to C in the supplemental material for all DESeq2 results. TCA, tricarboxylic acid.

We mapped reads from our host-environment metagenome data set to each cluster and used DESeq2 to identify those significantly enriched (adjusted *P* value of <1e−5) in each biome. Most strikingly, the same functional groups were enriched in both biomes, regardless of the grouping (i.e., COG functional category, KEGG pathway, or CAZy family); however, the gene clusters belonged to different microbial phyla (Fig. 6) (see the supplemental results in reference 15). For instance, nearly all COG categories for gene clusters belonging to *Proteobacteria* were environment enriched, while the same COG categories for clusters belonging to the *Firmicutes* and *Bacteroidetes* were host enriched. In contrast, functional groups of certain phyla were enriched in one biome, while different groups were enriched in the other, indicating within-phylum differences in functional contents and habitat distributions. For instance, *Fusobacteria* KEGG pathways were predominantly host enriched, but protein export, the bacterial secretion system, and aminoacyl-tRNA biosynthesis were environment enriched, indicating that these 3 pathways were more predominant in environment-enriched members of the *Fusobacteria* (Fig. 6B). Overall, these results suggest that both biomes select for these same microbial functions, but the microbes involved often differ at coarse taxonomic scales.

10.1128/mSystems.01045-20.4 TABLE S4 (A) All SGB BGCs identified by AntiSMASH and clustered by BiGSCAPE into clans and families. These data are shown in Fig. S10 of N. D. Youngblut, J. de la Cuesta-Zuluaga, G. H. Reischer, S. Dauser, et al. (bioRxiv, 2020, https://doi.org/10.1101/2020.06.05.135962). (B) All AMR markers identified by Abricate, summed by species. These data are shown in Fig. S9 at the URL mentioned above. Download Table S4, XLSX file, 1.0 MB. Copyright © 2020 Youngblut et al. 2020 Youngblut et al. This content is distributed under the terms of the Creative Commons Attribution 4.0 International license.

10.1128/mSystems.01045-20.5 TABLE S5 (A) DESeq2 results for testing of host versus environment enrichment of gene cluster abundances (50% sequence identity clustering) summed by COG functional category and phylum. Positive and negative log_2_-fold change values signify enrichment in host-associated and environment metagenomes, respectively. These data are shown in Fig. 6. (B) DESeq2 results for testing of host versus environment enrichment of gene cluster abundances (50% sequence identity clustering) summed by KEGG pathway and phylum. Positive and negative log_2_-fold change values signify enrichment in host-associated and environment metagenomes, respectively. These data are shown in Fig. 6. (C) DESeq2 results for testing of host versus environment enrichment of gene cluster abundances (50% sequence identity clustering) summed by CAZy family and phylum. Positive and negative log_2_-fold change values signify enrichment in host-associated and environment metagenomes, respectively. These data are shown in Fig. 6. (D) DESeq2 results for testing of Mammalia versus non-Mammalia enrichment of gene cluster abundances (50% sequence identity clustering) summed by COG functional category and phylum. Positive and negative log_2_-fold change values signify enrichment in Mammalia and non-Mammalia metagenomes, respectively. These data are shown in Fig. S15 of N. D. Youngblut, J. de la Cuesta-Zuluaga, G. H. Reischer, S. Dauser, et al. (bioRxiv, 2020, https://doi.org/10.1101/2020.06.05.135962). (E) DESeq2 results for testing of Mammalia versus non-Mammalia enrichment of gene cluster abundances (50% sequence identity clustering) summed by KEGG pathway and phylum. Positive and negative log_2_-fold change values signify enrichment in Mammalia and non-Mammalia metagenomes, respectively. These data are shown in Fig. S15 at the URL mentioned above. (F) DESeq2 results for testing of Mammalia versus non-Mammalia enrichment of gene cluster abundances (50% sequence identity clustering) summed by CAZy family and phylum. Positive and negative log_2_-fold change values signify enrichment in Mammalia and non-Mammalia metagenomes, respectively. These data are shown in Fig. S15 at the URL mentioned above. Download Table S5, XLSX file, 0.3 MB. Copyright © 2020 Youngblut et al. 2020 Youngblut et al. This content is distributed under the terms of the Creative Commons Attribution 4.0 International license.

We also assessed gene cluster enrichment in Mammalia versus non-Mammalia and found fewer significantly enriched features, which may be due to the smaller metagenome sample size or less pronounced partitioning of functional groups among biomes (see Fig. S15 and the supplemental results in reference 15). Still, we again observed that both biomes were enriched for the same microbial functions, but these belonged to different coarse taxonomic groups. To assess whether abundance estimations were substantially erroneous due to mismapping of metagenome reads to the gene clusters, we reran the analysis with stricter DIAMOND mapping parameters but observed similar findings, even though 48% fewer gene clusters were detected in any metagenome (see Fig. S16 in reference 15).

### Functional metagenome profiling benefits from our gene catalogue.

Finally, we integrated our gene catalogue into a custom HUMAnN2 database built from GTDB-r89 and found that this combined database substantially increased the mappability of reads from our multispecies metagenome data set (see Fig. S17 and the supplemental results in reference 15).

## DISCUSSION

Our MAG and gene cluster data sets, derived from 289 newly generated metagenomes from 180 vertebrate species, along with 544 metagenomes from 14 publicly available animal gut metagenome data sets, substantially helps to expand the breadth of cross-species gut metagenome comparisons (Fig. 1 and 5). While metagenomics is rapidly expanding in popularity (7), most analyses of metagenomic data suffer from a reliance on incomplete reference databases (20), which we show to be acutely problematic for the gut microbiomes of most vertebrates in our data set (Fig. 1). Grossly incomplete surveys of microbial diversity can lead to incorrect findings on community assembly in the vertebrate gut (21). Although our data set has only partially revealed this unknown diversity, it substantially improves reference database coverage at both the genome and gene levels and also provides an estimate of the incompleteness of existing reference databases.

A major contribution of this study is the extensive MAG collection that we generated by assembling the metagenomes of our multispecies data set together with 14 other animal gut metagenome data sets from understudied host species. This collection includes 1,184, 266, and 6 genomes from novel species, genera, and families, respectively (Fig. 2) (see Fig. S4 in reference 15). Moreover, we found little overlap (31%) between our MAG collection and the extensive human microbiome genome catalogue comprising the UHGG, which underscores its taxonomic novelty. We also showed substantial SGB prevalence across all 5 vertebrate taxonomic classes (see Fig. S5 in reference 15), indicating that our MAG collection is representative of microbes found across the vertebrate taxonomy. Our MAG collection, once combined with the GTDB (22), improved our ability to classify reads in our multispecies metagenome data set (see Fig. S6 in reference 15), which is critical for accurately assessing gut microbiome diversity across vertebrates. Although MAGs have been criticized for their incompleteness and potentially high prevalence of misassemblies (23), we note that (i) the overall completeness of our MAGs was rather high (90% median completeness), (ii) complete genomes are not required for accurate taxonomic profiling (24), and (iii) the prevalence of misassemblies among MAGs is likely quite low when using state-of-the-art assembly and binning approaches (25). Still, researchers who may utilize this set of MAGs should use caution when analyzing individual single nucleotide polymorphisms (SNPs), plasmids, genomic islands, or other potentially missing or misassembled genomic features (26).

We investigated the distribution of our MAGs across environment and host biomes to elucidate the diversity of host-microbe symbiosis in the vertebrate gut. Microbe-host symbiosis spans the continuum from free-living microbes that can simply survive passage through the host gut to obligate symbioses (27). Therefore, MAGs enriched in the environment versus the host would indicate a weak association, while the opposite enrichment would suggest a more obligate symbiosis. We provide evidence of host specificity for the majority of SGBs, while a few *Proteobacteria* and *Actinobacteria* SGBs were environment enriched. When considering just host-associated metagenomes, these env-SGBs were generally enriched in nonmammals (Fig. 2 and 3) (see Fig. S8 in reference 15). This is consistent with the hypothesis that mixed-mode transmission, especially between environmental sources and hosts, is more commonplace in nonmammalian gut microbiome community assembly than in mammals (28).

Our trait-based analysis of SGBs supports the notion that host-enriched taxa are adapted for a symbiotic lifestyle, while environment-enriched taxa are adapted for a free-living or facultative symbiosis lifestyle (Fig. 3). For instance, anaerobes comprised almost all host-enriched SGBs, while environment-enriched SGBs were aerobes or facultative anaerobes and generally motile, which could be highly beneficial for transmission between the environment and gut biomes. Indeed, a recent directed evolution experiment showed that selecting for interhost migration can generate bacterial strains with increased motility (29), and a trait-based study of the human infant gut microbiome showed that later stages of succession are dominated by taxa adapted to the anoxic gut (30).

By assessing SGB enrichment in Mammalia versus non-Mammalia metagenomes, we elucidated the specificity of host-microbe symbioses in the gut across large evolutionary distances. More SGBs were enriched in mammals than in nonmammals (Fig. 2) (see Fig. S8 in reference 15), as we observed in our previous 16S rRNA assessment of these vertebrate clades (12). Few traits differed among SGBs enriched in either biome (see Fig. S8 in reference 15), which may indicate that the traits assessed are similarly required for adaptation to each host clade, even at this coarse evolutionary scale.

Vertebrates both play a critical role in the spread of antimicrobial resistance and also have been sources of novel antibiotics and other natural products (18, 31). We investigated BGC and AMR diversity in our MAG collection and observed a high diversity of BGC products, but very few of the BGCs clustered into families with experimentally characterized BGCs from the MIBiG database (see Fig. S9 and S10 in reference 15). This contrasts with findings that only ∼10% of BGCs in the human microbiome are uncharacterized (32), which is likely due to the limited study of natural products in the gut microbiome of nonhuman vertebrates (33, 34). We found NRPS-producing BGCs to be prevalent among the *Firmicutes* SGBs, which is similar to a recent assessment of 501 genomes from rumen isolates in which thousands of BGCs were identified (35). Still, RiPPs were most prevalent across all vertebrate clades, which expands upon observations of the high prevalence of this BGC class in the gut microbiome of humans (Fig. 2 and 4) (32).

By combining our AMR marker screen with our SGB biome enrichment analysis, we were able to characterize how AMR is associated with various degrees of symbiosis (see Fig. S9 in reference 15), which is important for understanding AMR reservoirs (18, 36). Our findings indicate that the AMR reservoir may be greater for free-living and facultatively symbiotic taxa than for microbes with stronger host associations (see Fig. S9 in reference 15). Indeed, some of the most abundant AMR markers were associated with metal resistance (e.g., *ruvB*, *tupC*, and *arsT*), which may reflect a lifestyle in which the microbe is exposed to environmental sources of metals (37, 38).

While MAGs provide a powerful means of investigating species- and strain-level diversity within the vertebrate gut microbiome, the approach is limited to only relatively abundant taxa with enough coverage to reach adequate assembly contiguity (39). Our gene-based assembly approach allowed us to greatly expand the known gene catalogue of the vertebrate gut microbiome beyond just the abundant taxa, with a total of >150 million nonredundant coding sequences generated, comprising 88 bacterial and 11 archaeal phyla (Fig. 5). In comparison, recent large-scale metagenome assemblies of the gut microbiome from chickens, pigs, rats, and dogs have generated 7.7 million, 9.04 million, 7.7 million, 5.1 million, and 1.25 million nonredundant coding sequences, respectively (8, 16, 40, 41). It is also illustrative to consider that a recent large-scale metagenome assembly of cattle rumen metagenomes generated 69,678 nonredundant genes involved in carbohydrate metabolism (9), while our gene collection comprised substantially more CAZy-annotated gene clusters (*n *= 87,573), even after collapsing at 50% sequence identity. The increased mappability that we achieved across all 5 vertebrate clades when incorporating our gene catalogue into our functional metagenome profiling pipeline demonstrates how our gene collection will likely aid future vertebrate gut metagenome studies (see Fig. S17 in reference 15).

Our assessment of gene cluster abundances in metagenomes from environment- and host-associated biomes illuminates how microbiome functioning and taxonomy are distributed across the free-living to obligate symbiont spectrum. Most notably, nearly all prominent functional groups were enriched in both the environment- and host-associated biomes, but the specific gene clusters belonged to different taxonomic groups in each biome (Fig. 6). For instance, almost all abundant CAZy families were enriched in both the environment and host biomes, but the environment was dominated by *Proteobacteria*, while *Firmicutes*, *Bacteroidetes*, and *Actinobacteria* gene clusters comprised most host-enriched CAZy families. This suggests that the same coarse-level functional groups are present across the free-living to obligate microbe-vertebrate symbiosis lifestyles, but coarse-level taxonomy strongly differs across this spectrum. This pattern largely remained true when we compared enrichment between the Mammalia and nonmammals, suggesting that taxonomic differences prevail over functional differences in regard to host specificity, at least over broad-scale vertebrate evolutionary distances. While comparing function to taxonomy is challenging due to differing levels of resolution, we do not believe that our findings are simply due to using functional groupings that are coarser than taxonomy, given that (i) we assessed multiple functional groupings (COG, KEGG, and CAZy), which all showed similar patterns, even though they differ in functional resolution, and (ii) we assessed taxonomy at the very coarse phylum level but still found stark taxonomic differences across biomes.

In conclusion, our large-scale metagenome assembly of both MAGs and coding sequences from a diverse collection of vertebrates substantially expands the known taxonomic and functional diversity of the vertebrate gut microbiome. We have demonstrated that both taxonomic and functional metagenome profiling of the vertebrate gut is improved by our MAG and gene catalogues, which will aid future investigations of the vertebrate gut microbiome. Moreover, our collection can help guide natural product discovery and bioprospecting of novel carbohydrate-active enzymes, along with modeling AMR transmission among reservoirs. By characterizing the distribution of MAGs and microbial genes across environment and host biomes, we gained insight into how taxonomy and function differ along the free-living to obligate symbiosis lifestyle spectrum. We must note that our metagenome assembly data set is biased toward certain animal clades, which likely impacts these findings. As metagenome assembly becomes more commonplace for studying the vertebrate gut microbiome, bias toward certain vertebrates (e.g., humans) will decrease and thus allow for a more comprehensive reassessment of our findings.

## MATERIALS AND METHODS

### Sample collection.

Sample collection was performed as described previously by Youngblut and colleagues (12). Table S1A in the supplemental material shows the dates, locations, and additional metadata for all samples collected. All fecal samples were collected in sterile sampling vials, transported to a laboratory, and frozen within 8 h. DNA extraction was performed with the PowerSoil DNA isolation kit (MoBio Laboratories, Carlsbad, CA, USA).

### “Multispecies” vertebrate gut metagenomes.

Metagenome libraries were prepared as described previously by Karasov and colleagues (42). Briefly, 1 ng of input gDNA was used for Nextera Tn*5* tagmentation. BluePippin was used to restrict fragment sizes to 400 to 700 bp. Barcoded samples were pooled and sequenced on an Illumina HiSeq3000 instrument with 2-by-150 paired-end sequencing. Read quality control (QC) was described previously (see the supplemental methods in reference 15).

Post-QC reads were taxonomically profiled with Kraken2 and Bracken v.2.2 (43) against the Struo-generated GTDB-r89 Kraken2 and Bracken databases (20). HUMAnN2 v.0.11.2 (44) was used to profile genes and pathways against the Struo-generated HUMAnN2 database created from GTDB-r89.

### Publicly available animal gut metagenomes.

Published animal gut metagenome reads were downloaded from the Sequence Read Archive (SRA) between May and August 2019. Table S1B lists all included studies. We selected studies with Illumina paired-end metagenomes from gut contents or feces. MGnify samples were downloaded from the SRA in October 2019 (Table S1C). Read quality control was described previously (see the supplemental methods in reference 15).

### Pipeline for metagenome assembly of genomes.

Assemblies were performed on a per-sample basis, with reads subsampled via seqtk v.1.3 to ≤20 million read pairs. The details of the assembly pipeline were described previously (see the supplemental methods in reference 15).

A multilocus phylogeny of all SGB representatives was inferred with PhyloPhlAn v.0.41 (45). Secondary metabolites were identified with AntiSMASH v.5.1.1 (46) and DeepBGC v.0.1.18 (47) and then characterized with BiGSCAPE (19). Abricate was used to identify antimicrobial resistance genes. We used Krakenuniq v.0.5.8 (48) for estimating the abundance of MAGs in metagenome samples. (Details can bee found in the supplemental methods in reference 15.)

### Pipeline for metagenome assembly of genes.

Assemblies were performed on a per-sample basis, with reads subsampled via seqtk v.1.3 to ≤20 million pairs. We used PLASS v.2.c7e35 (14) and Linclust (mmseqs v.10.6d92c) (13) to assemble and cluster contigs. A full description was reported previously (see the supplemental methods in reference 15). DESeq2 (49) was used to estimate the enrichment of MAGs and gene clusters in metagenomes from host and environment biomes.

### Data availability.

The raw sequence data are available from the European Nucleotide Archive under study accession number PRJEB38078. Fasta files for the 5,596 nonredundant MAGs, 1,522 SGBs, and gene clusters (50, 90, and 100% sequence identity clustering) can be found at http://ftp.tue.mpg.de/ebio/projects/animal_gut_metagenome_assembly/, along with GenBank files for all BGCs. The code used for processing the data can be found at https://github.com/leylabmpi/animal_gut_metagenome_assembly.
